# Publisher Correction: Transcriptomic alterations during ageing reflect the shift from cancer to degenerative diseases in the elderly

**DOI:** 10.1038/s41467-019-10559-5

**Published:** 2019-05-31

**Authors:** Peer Aramillo Irizar, Sascha Schäuble, Daniela Esser, Marco Groth, Christiane Frahm, Steffen Priebe, Mario Baumgart, Nils Hartmann, Shiva Marthandan, Uwe Menzel, Jule Müller, Silvio Schmidt, Volker Ast, Amke Caliebe, Rainer König, Michael Krawczak, Michael Ristow, Stefan Schuster, Alessandro Cellerino, Stephan Diekmann, Christoph Englert, Peter Hemmerich, Jürgen Sühnel, Reinhard Guthke, Otto W. Witte, Matthias Platzer, Eytan Ruppin, Christoph Kaleta

**Affiliations:** 1 Research Group Medical Systems Biology, Institute of Experimental Medicine, Christian-Albrechts-University Kiel, D-24105 Kiel, Germany; 2 Jena University Language and Information Engineering Lab, Friedrich-Schiller-University Jena, D-07743 Jena, Germany; 3 GerontoSys JenAge Consortium, D-07745 Jena, Germany; 4 Genome Analysis Lab, Leibniz Institute on Aging–Fritz-Lipmann-Institute, D-07745 Jena, Germany; 5 Hans Berger Department of Neurology, Jena University Hospital, D-07747 Jena, Germany; 6 Systems Biology and Bioinformatics Group, Leibniz Institute for Natural Product Research and Infection Biology–Hans-Knöll-Institute, D-07745 Jena, Germany; 7 Biology of Ageing Lab, Leibniz Institute on Aging–Fritz-Lipmann-Institute, D-07745 Jena, Germany; 8 Molecular Genetics Lab, Leibniz Institute on Aging–Fritz-Lipmann-Institute, D-07745 Jena, Germany; 9 Imageing Facility, Leibniz Institute on Aging–Fritz-Lipmann-Institute, D-07745 Jena, Germany; 10 Integrated Research and Treatment Center, Center for Sepsis Control and Care (CSCC), Jena University Hospital, D-07747 Jena, Germany; 11 Network Modeling, Leibniz Institute for Natural Product Research and Infection Biology–Hans Knöll Institute, D-07745 Jena, Germany; 12 Institute for Medical Informatics and Statistics, Christian-Albrechts-University Kiel, D-24105 Kiel, Germany; 13 Energy Metabolism Laboratory, Swiss Federal Institute of Technology (ETH) Zurich, Schwerzenbach/Zürich, CH-8603 Switzerland; 14 Department of Bioinformatics, Friedrich-Schiller-University Jena, D-07743 Jena, Germany; 15 Laboratory of Neurobiology, Scuola Normale Superiore, University of Pisa, I-56100 Pisa, Italy; 16 Molecular Biology Lab, Leibniz Institute on Aging–Fritz-Lipmann-Institute, D-07745 Jena, Germany; 17 Faculty of Biology and Pharmacy, Friedrich-Schiller-University Jena, D-07743 Jena, Germany; 18 Biocomputing Lab, Leibniz Institute on Aging–Fritz-Lipmann-Institute, D-07745 Jena, Germany; 19 Department of Computer Science and Center for Bioinformatics and Computational Biology, University of Maryland, College Park, MD 20742 USA

**Keywords:** Computational biology and bioinformatics, Ageing, Genetic association study, Cancer

Correction to: *Nature Communications* 10.1038/s41467-017-02395-2, published online 30 January 2018.

The original version of this Article contained an error in the spelling of the author Jule Müller, which was incorrectly given as Julia Müller. Additionally, in Fig. 4a, the blue-red colour scale for fold change in ageing/disease regulation included a blue stripe in place of a red stripe at the right-hand end of the scale. These errors have been corrected in both the PDF and HTML versions of the Article.

