# Supplementary material for: Transcriptomic alterations during ageing reflect the shift from cancer to degenerative diseases in the elderly
Source: Nat Commun. 2018 Jan 30;9:327. doi: 10.1038/s41467-017-02395-2 (PMC5790807; doi:10.1038/s41467-017-02395-2)
Supplement: Supplementary file 1 — Supplementary Information [file 41467_2017_2395_MOESM1_ESM.pdf]

Supplementary Figures

Supplementary Figure 1

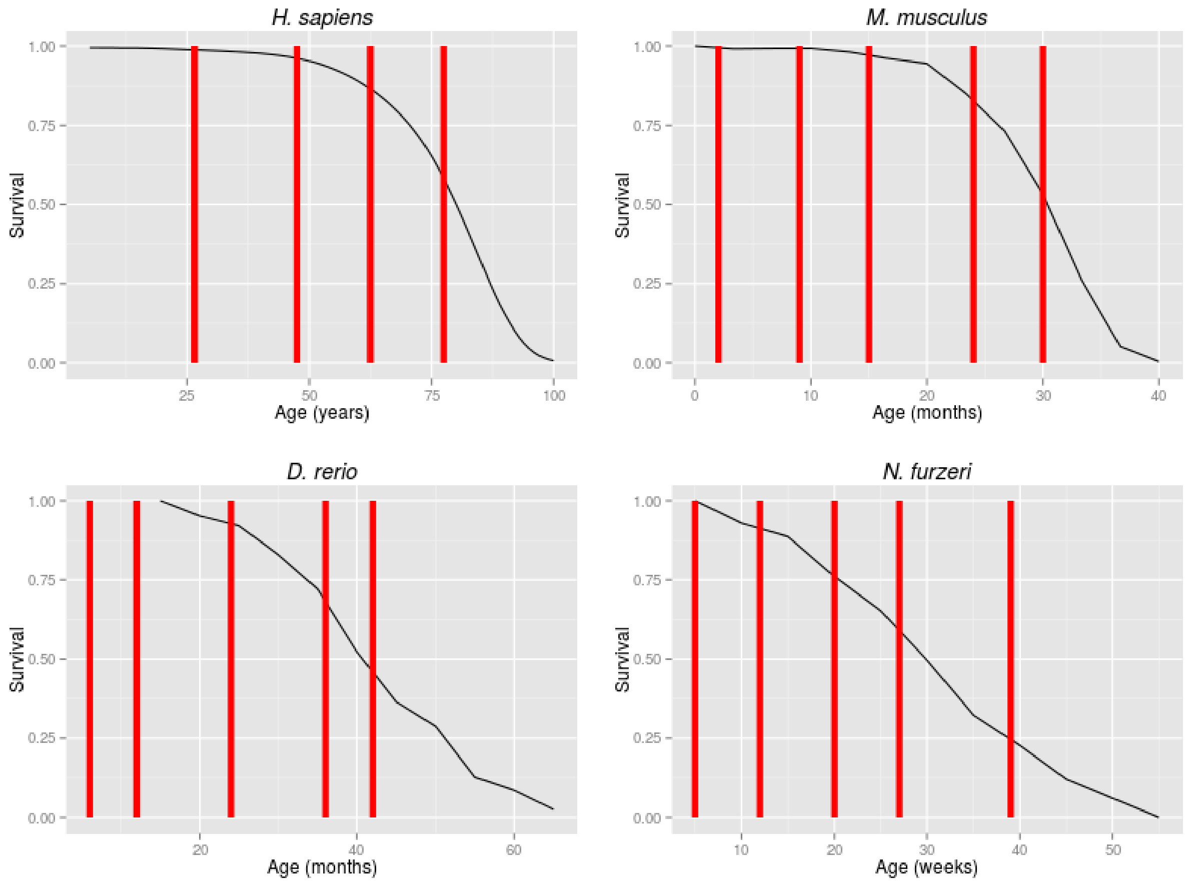

Survival curves of species. Red bars indicate time points for which data was generated in our study. Data for *Homo sapiens* has been obtained from the Human Life-Table Database (http://www.lifetable.de), data for Germany (years 2006-2008), male survival. Sampling time points: 24-29 years, 45-50 years, 60-65 years and 75-80 years (mid-points displayed). Data for *Mus musculus* has been obtained from Kunstyr and Leuenberger [67], male data, life-table recording started at 50 days of age, n=296. Sampling time points: 2 months, 9 months, 15 months, 24 months and 30 months. Data for *Danio rerio* has been obtained from Gerhard, et al. [68], outbred animals, average of survival in two tanks, life-table recording started at 17 months of age, n=77. Sampling time points: 6 months, 12 months, 24 months, 36 months and 42 months. Data for *Nothobranchius furzeri* has been obtained from Tozzini, et al. [69], data of MZM 04/10 strain, life-table recording started at 5 weeks of age, n= 113. Sampling time points: 5 weeks, 12 weeks, 20 weeks, 27 weeks and 39 weeks.

Supplementary Figure 2

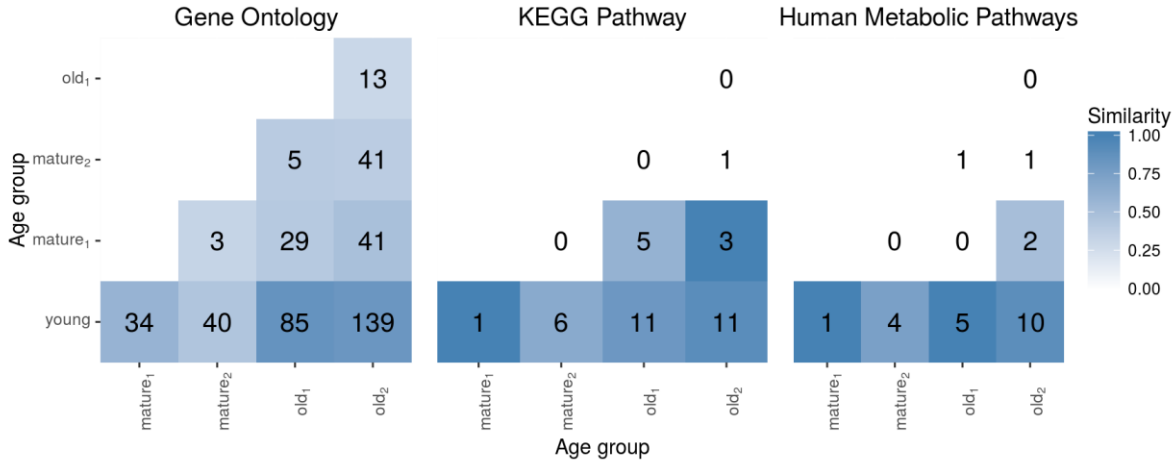

Number of processes with ageing-associated changes between pairs of age-groups. The shading ("Similarity") indicates which fraction of differentially regulated processes were also differentially regulated in the general ageing signature (comparison between the young and the two old age groups). We found a total of 492 cases of significant differential regulation of processes in the comparison between individual age groups. Of these cases, 324 (66%) showed the same direction of regulation like the ageing signature while six cases (1.2%) showed a regulation opposing the ageing signature. The remaining cases corresponded to processes that were not significantly differentially regulated in the cross-species comparison.

Supplementary Figure 3

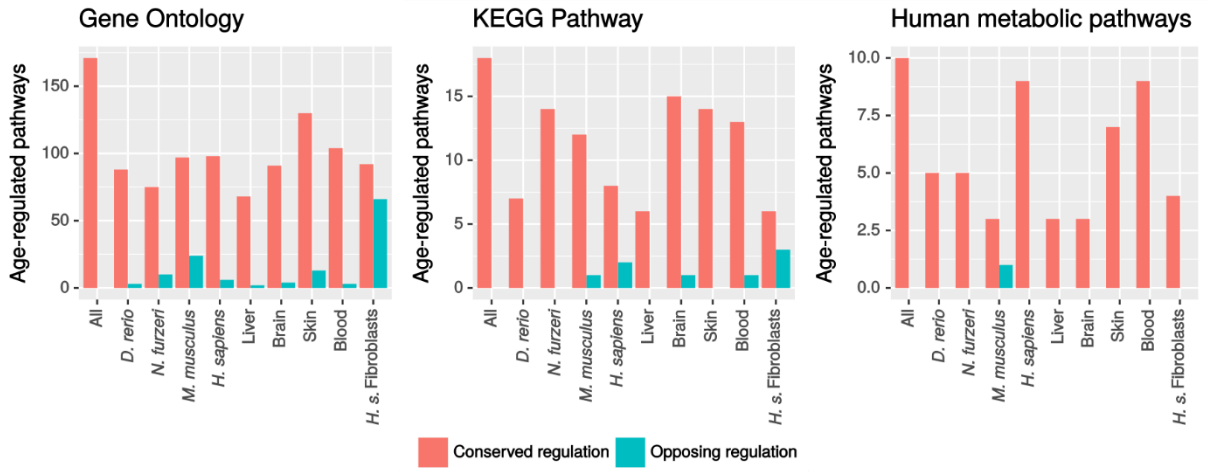

Conservation of ageing-associated transcriptional changes in individual species and tissues. For each ontology, the number of significantly differentially regulated processes with ageing for the specific subset of data (i.e. from a species or a tissue) is shown for cases where ageing-regulation is concordant with the cross-species regulation (red) and where processes show opposite changes compared to cross-species regulation (turquoise). For further information see also Supplementary Note S3.1.

Supplementary Figure 4

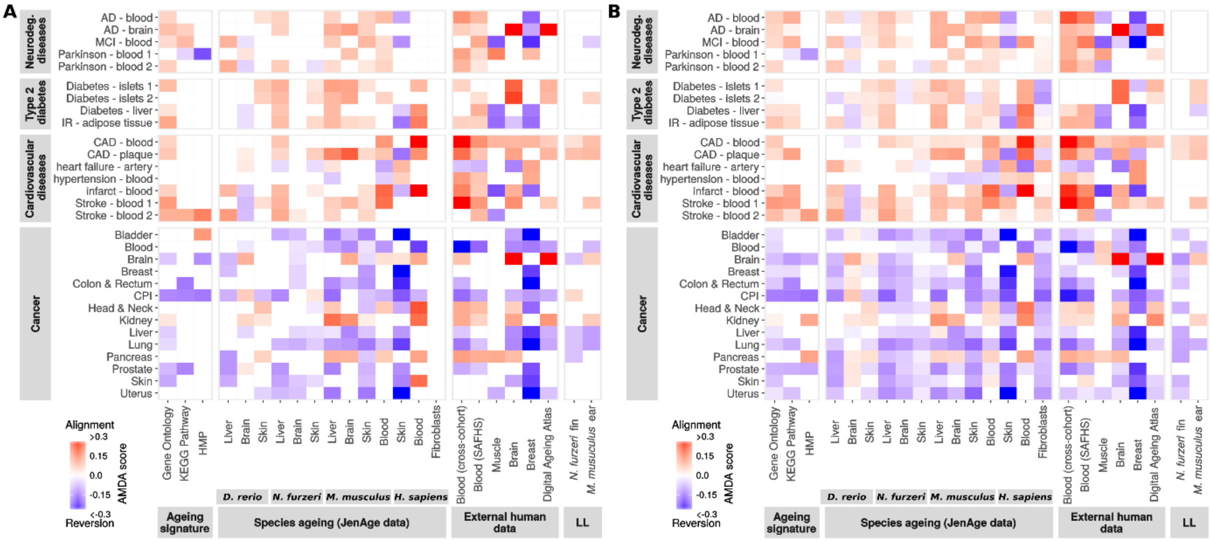

Influence of senescence-associated processes and cellular proliferation on ageing-mediated disease alignment. To assess the role of cellular senescence in ageing-mediated disease alignment, we determined AMDA scores between ageing and disease data sets after removing data for all genes associated with cellular senescence. As reference for genes playing a role in cellular senescence, we used the list of significantly differentially expressed genes of cell cultures transitioning into senescence (fibroblast data, 13438 genes, sub-panel A) and a list of all genes associated with gene ontology processes containing the term "proliferation" (2125 genes, sub-panel B). For both gene lists, we found a strong alignment of ageing-associated gene expression changes with the signature of degenerative diseases and a reversal of the gene expression signature of cancer. These results suggest that while cellular senescence is an important driver of ageing-associated pathologies[70], also non-senescence-associated processes contribute to the pronounced shifts in the ageing transcriptome relative to disease signatures.

Supplementary Note 1: Epidemiology of ageing-associated diseases

Mortality data

Data on causes of mortality for the US population in 2010 was downloaded from the website of the Center for Disease Prevention and Control (http://www.cdc.gov/). Since CDC does not provide single-year population sizes for the different age groups beyond the age of 85, the corresponding data was obtained from the 2010 US census. We used disease data classified according to the 113 causes list of CDC. To obtain the contribution of different diseases to total mortality, we only considered "rankable" causes of death according to the CDC site. These are diseases that can appear in the top 15 causes of death and were assumed to be non-redundant. This distinction was necessary since some causes of death in the CDC 113 list are included in others (e.g. different neoplasms that also figure individually in the list are included in the general term "Malignant neoplasms (C00-C97)"). Census data for people older or equal 100 years was combined into the age group "100+" to match CDC data. Causes of death were grouped into the categories "Cancer", "Cardiovascular diseases", "Neurodegenerative diseases", "Diabetes" (including type I and type II, type I accounting for 4.4% of cases in the US in 2012 according to CDC), "External causes" and "Other causes" as follows (ICD-10 codes of the International Statistical Classification of Diseases and Related Health Problems of the World Health Organization in brackets):

Cancer:

- Malignant neoplasms (C00-C97)
- In situ neoplasms, benign neoplasms and neoplasms of uncertain or unknown behavior (D00-D48)

Cardiovascular diseases:

- Diseases of heart (I00-I09,I11,I13,I20-I51)
- Essential hypertension and hypertensive renal disease (I10,I12,I15)
- Cerebrovascular diseases (I60-I69)
- Atherosclerosis (I70)
- Aortic aneurysm and dissection (I71)

Neurodegenerative diseases:

- Parkinson's disease (G20-G21)
- Alzheimer's disease (G30)

Diabetes:

- Diabetes mellitus (E10-E14)

External causes:

- Accidents (unintentional injuries) (V01-X59,Y85-Y86)
- Intentional self-harm (suicide) (*U03,X60-X84,Y87.0)
- Assault (homicide) (*U01-*U02,X85-Y09,Y87.1)
- Legal intervention (Y35,Y89.0)
- Operations of war and their sequelae (Y36,Y89.1)
- Complications of medical and surgical care (Y40-Y84,Y88)

Other causes: all other items

Fractional mortality contribution was obtained by dividing, for each single-year age group, the number of deaths attributable to each disease category by total mortality across all categories.

Incidence of cancer, cardiovascular disease, dementia and type 2 diabetes

Cancer incidence in the USA for the year 2010 from the Surveillance, Epidemiology and End Results Program (SEER, http://seer.cancer.gov/) for the SEER 18 registries covering 18 regions of the US was obtained from the SEER database. The SEER database contains data about diagnosis of cancer for 34

different cancer sites and 19 age groups, $a_1 \ldots a_{19}$, spanning 0 to over 84 years in the intervals 0 – 12 months, 1 – 4 years, 5 – 9 years, 10 – 14 years, ..., 80 – 84 years and above 84 years from different regions of the US (grouped into registries). We considered only data for 2010 since SEER does not provide population data for the age groups beyond 85, while detailed age information for all age groups is available for this year based on the 2010 US census matched to the regions covered by SEER. Population numbers of the SEER 18 registries were obtained from the Census data base (factfinder.census.gov, Table PCT12). Based on the SEER raw data, we obtained cancer incidence additionally including the age groups 85 - 89 years, 90 – 94 years, 95 – 99 years and above 100 years by counting, for each age group, the number of persons which had a cancer diagnosed for the first time (information available from SEER) divided by the total population count from the census data.

Epidemiological data for neurodegenerative diseases in form of dementia was obtained from Rocca, et al. [1] (incidence was averaged across all birth cohorts from Rochester, Minnesota, USA), data for incidence of cardiovascular disease was obtained from Driver, et al. [2] and data for type 2 diabetes from Thunander, et al. [3].

A note on type 2 diabetes incidence

In contrast to the other diseases, reliable data on the change of type 2 diabetes incidence across age groups is difficult to obtain due to diabetes risk being strongly influenced by obesity[4] and the prevalent underdiagnosis of type 2 diabetes[5]. In particular, data from Field et al.[4] shows that the risk of developing diabetes in case of obesity is often several times the risk of colon cancer, heart disease or stroke (Supplementary Table 1). Thus, the contribution of the severe rise of obesity in recent decades[6] is expected to strongly influence cross-sectional data on the incidence of type 2 diabetes. Indeed, strong differences in incidence of type 2 diabetes between age groups from different birth cohorts has been noted in comparison of birth cohort data and cross-sectional data[7]. Notably, while cross-sectional data from the US across birth cohorts indicated a plateau or even declining type 2 diabetes prevalence for the oldest-old, separating the population into birth cohorts showed rising prevalence up to the oldest age groups for the individual age groups[7]. The strong influence of the rise of obesity in these observation is also reflected in the comparison of data from the US (with declining diabetes prevalence for older age groups and an average body mass index (BMI) of 28.8 in the adult population according to the WHO) with data from North European countries (average BMI of 25.3 for Denmark and 25.8 for Sweden), for which rising incidence has been reported up to the oldest age groups[3,8]. Importantly, the decline in cancer incidence is also observed in data stratified for birth cohorts in the US[9].

Another contributing factor are observations that type 2 diabetes is often undiagnosed, with undiagnosed cases of diabetes estimated to contribute up to 46% of total disease cases in the above 75 year olds[5]. Along these lines, diabetes is also underreported on death certificates with only 39% of cases with diabetes actually mentioned on death certificates[10]. These observations have a strong influence on the reliability of type 2 diabetes incidence that reflects all actual disease cases (i.e. no undiagnosed cases) and is not biased by the recent global rise in obesity. Longitudinal follow up data can overcome these issues, yet longitudinal data over long follow up times is scarcely available. However, there is data from the Framingham Heart Study available who measured, amongst others, blood glucose levels as one parameter during follow-ups[11]. Increasing blood glucose levels are among the first changes observed before the actual onset of type 2 diabetes and have been shown to strongly increase up to the highest age groups (95 years) in lifespan-stratified patient data from the

Framingham cohort[11]. This indicates that, besides obesity as a much stronger contributor compared to the other diseases, also age up to the highest age groups drives type 2 diabetes incidence.

Supplementary Table 1 Obesity-associated risk of major ageing-associated diseases. Data taken from [4]. Risks with reference to individuals with BMIs in the range of 18.5 to 21.9. 95% confidence intervals are indicated in brackets. A BMI between 18.5 and 25 is considered normal weight and a BMI of 25 to 30 as overweight according to the World Health Organization.

BMI, group	Diabetes risk	Colon cancer risk	Heart disease risk	Stroke risk
22-24.9 (female)	2.2 (1.7-3.1)	1.3 (1.0-1.7)	1.2 (1.1-1.4)	0.9- (0.7-1.1).
22-24.9 (male)	1.8 (1.2-2.7)	1.9 (1.1-3.1)	1.1 (1.0-1.4)	1.1 (0.8-1.6)
25-29.9 (female)	8.1 (6.1-10.7)	1.3 (1.0-1.7)	1.5 (1.4-1.7)	1.0 (0.8-1.3)
25-29.9 (male)	5.6 (3.7-8.4)	2.0 (1.2-3.3)	1.7 (1.4-2.0)	1.3 (0.9-1.9)

The Armitage-Doll model for modelling cancer incidence

The Armitage-Doll model assumes that cancer arises as series of consecutive mutation events that are required for a normal cell to become malignant[12], referred here to as malignant transformation steps. Assuming seven mutations to be required for such a transformation, the incidence of a specific cancer *s* at age *t*, c_t^s, can be derived as[12]

$$c_t^s = kp_1p_2p_3p_4p_5p_6p_7t^6$$

with *k* being a constant and $p_1, \ldots, p_7$ the probabilities of the individual mutations. These were assumed to be constant across age. Through logarithmic transformation we obtained

$$\log c_t^s = \log(kp_1p_2p_3p_4p_5p_6p_7) + 6 \cdot \log t$$

Thus, the logarithm of cancer incidence rises as a sum of a constant plus six times the logarithm of time.

As already noted by Armitage and Doll, the logarithmic incidence of some cancers appeared to rise with a different rate with time, termed here the malignant transformation rate c_{trans}^s for a cancer *s*, which corresponds to the rate at which premalignant cells (successfully) acquire additional mutations until full malignant transformation (i.e. a clinically detectable tumor). Hence, in this case, cancer incidence growth can be modelled as

$$c_t^s = kp_1p_2p_3p_4p_5p_6p_7t^{c_{trans}^s}$$

Now, if we assume the individual transition probabilities to stay relatively constant between two age-groups a_i and a_{i+1}, we can estimate the malignant transformation rate c_{trans}^s by

$$c_{a_i}^s = kp_1p_2p_3p_4p_5p_6p_7mp(a_i)^{c_{trans}^s}$$

$$c_{a_{i+1}}^{s} = kp_1 p_2 p_3 p_4 p_5 p_6 p_7 mp(a_{i+1})^{c_{trans}^{s}}$$

$$c_{growth}^{s} = \frac{\log(c_{a_{i+1}}^{s}) - \log(c_{a_i}^{s})}{\log(mp(a_{i+1})) - \log(mp(a_i))}$$

with *mp(a*$_i$*)* being a function that returns the mid-point of the age group a_i. Since this growth rate represents a local estimate depending on the choice of age-groups, we referred to it as $c_{trans}^{s}(a_i, a_{i+1})$, the malignant transformation rate between the age groups a_i and a_{i+1}. Please note that the malignant transformation rate has been referred to as log-log acceleration of cancer incidence previously in the context of the analysis of a selected number of cancer types [13]. Based on the Armitage-Doll model, $c_{trans}^{s}(a_i, a_{i+1})$ can be interpreted as the rate at which precancerous cells (successfully) acquire mutations and proliferate on their way to a clinically detectable tumor in the time that passes between the mid-points of the age groups a_i and a_{i+1}. Hence, increases of this rate can be caused by an increased likelihood of acquiring cancerous mutations (e.g. through mutagen exposure), a reduced efficiency of molecular mechanisms repairing potentially carcinogenic mutations (e.g. DNA damage response) or a reduced efficiency of processes clearing precancerous cells (e.g. immunosurveillance).

Malignant transformation rates in humans

For malignant transformation rates, we only considered age groups provided by SEER since for these age groups detailed data across all 34 cancer sites is available for the years 1975 to 2012 from the SEER 9 registries. We averaged cancer incidence per 100,000 persons across all incidences reported from 1975 to 2012, yielding the cancer incidence of each cancer site *s* in each age group a_i, $c_{a_i}^{s}$.

Malignant transformation rates $c_{trans}^{s}(a_i, a_{i+1})$ between each pair of adjacent age groups a_i and a_{i+1} for cancer site *s* were determined by fitting of an Armitage-Doll model by

$$c_{trans}^{s}(a_i, a_{i+1}) = \frac{\log(c_{a_{i+1}}^{s}) - \log(c_{a_i}^{s})}{\log(mp(a_{i+1})) - \log(mp(a_i))}$$

where *mp(a*$_i$*)* is a function that returns the mid-point (in years) of the age group a_i. For the age group "85+" years a mid-point of 87.5 years was chosen. Please note that this mid-point cannot be reliably estimated since this group also contains individuals older than 90 years. Based on the 2010 census and assuming all people older than 100 to be of age 100 (the census only provides ages in 5 year age groups beyond 100), the average age in this group is 89 years.

Malignant transformation rates in mice

We estimated malignant transformation rates for mice treated with different doses of the carcinogen 2-acetylaminofluorene reported in the ED01 study[14]. This data set represents one of the largest studies in which the carcinogenic potential of different doses of 2-acetylaminofluorene was analyzed in a cohort of 25,000 mice. We only considered data from mice that were not sacrificed. Age-specific changes in cancer incidence growth have previously been analyzed based on this data set also reporting decreasing cancer incidence rates with age[15]. The claims of this work, however, were later retracted due to an error in the database[16]. The corrected data set was kindly provided by Charles Harding, who also provided a script for determining malignant transformation rates based on this

data set. Based on the corrected data set, we determined age-specific locally smoothed hazard rates for cancer incidence for mice not sacrificed during the study and diagnosed with cancer as cause of death using the R “muhaz” package (http:// cran.r-project.org/package=muhaz). Based on cancer incidence rates, we determined malignant transformation rates as described above for different doses of treatment with 2-acetylaminofluorene (Supplementary Fig. 5). We used the grid points (n=50, life span varied depending on treatment) returned by the smoothing function of “muhaz” as individual age groups. To obtain 95% confidence intervals for the malignant transformation rate estimates, we resampled the data for each dose 1000 times with replacement. In similarity to the results obtained for human populations, we observed the highest malignant transformation rates at middle-ages around 500 days (approx. 17 months) for mice treated with different doses of the carcinogen. At higher ages malignant transformation rates declined. Thus, also in mice cancer incidence growth decelerates at older ages.

8

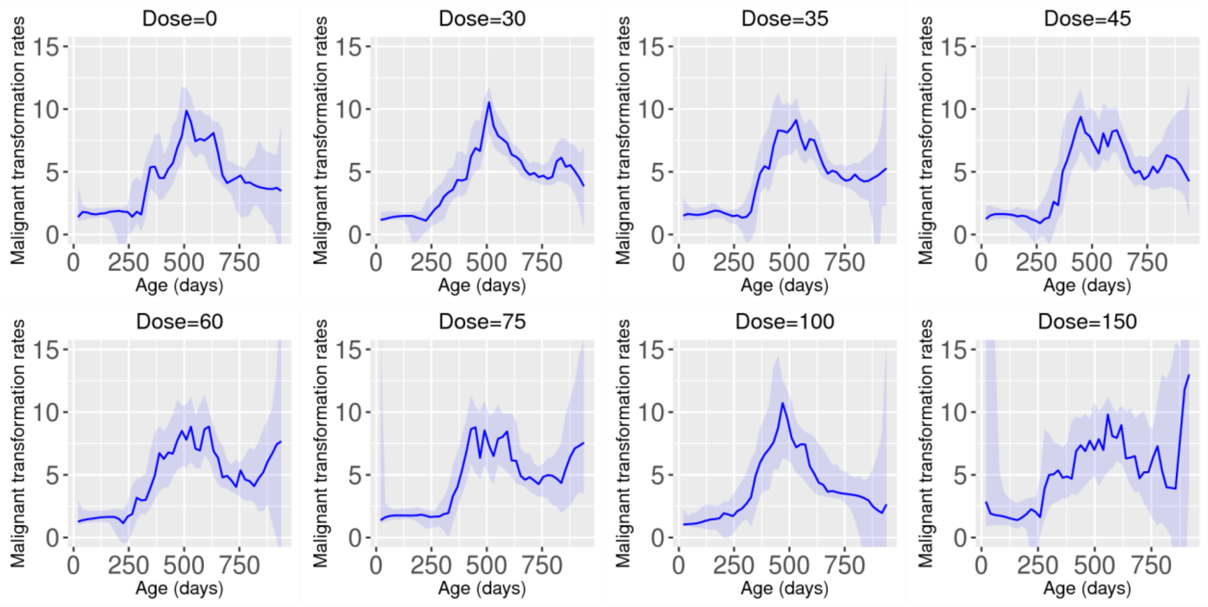

Supplementary Figure 5 Age-specific malignant transformation rates for several cohorts of mice treated with different doses of 2-acetylaminofluorene displayed in the header of each plot (dose in parts per million). The light blue area corresponds to the 95% confidence interval for the estimates based on resampling. For clarity, malignant transformation rates and confidence intervals are only displayed for a range of 0 to 15 on the y-axis.

Supplementary Note 2: Data sets

JenAge ageing datasets

Supplementary Table 2 Sample overview. The age for each sample is indicated in brackets behind the number of samples. The total number of data sets ("n") as well as the number of individuals from which samples have been obtained ("i") is indicated (see Supplementary Data 1, sheet "Sample overview" for more information). Numbers are provided separately for the cross-sectional design ("CS") and the longitudinal design ("LL"). Footnotes behind the tissue indicate works in which the corresponding data set was analyzed in the context of single-species analyses: Reichwald, et al. [17], Baumgart, et al. [18], Frahm, et al. [19], Marthandan, et al. [20]. Abbreviations: w – weeks, m – months, y – years, PD – population doublings (measure of age in cell cultures).

Species	Tissue	Number of samples and age				
		Young	$Mature_1$	$Mature_2$	Old_1	Old_2
Danio rerio TüAB n=73, i=45	**Total(CS)**	**14 (6m)**	**14 (12m)**	**15 (24m)**	**15 (36m)**	**15 (42m)**
	Brain(CS)	5	5	5	5	5
	Liver(CS)	4	4	5	5	5
	Skin(CS)	5	5	5	5	5
Nothobranchius furzeri MZM-0410 CS: n=75, i=33 LL: n=90, i=45	**Total (CS)**	**15 (5w)**	**15 (12w)**	**15 (20w)**	**15 (27w)**	**15 (39w)**
	Brain(CS)[17]	5	5	5	5	5
	Liver(CS) [17]	5	5	5	5	5
	Skin(CS) [17]	5	5	5	5	5
	Fin (LL)[18]	-	45 (10w)	45	-	-
Mus musculus C57BL/6J CS: n=112, i=88 LL: n=16, i=8	**Total(CS)**	**22 (2m)**	**23 (9m)**	**23 (15m)**	**21 (24m)**	**23 (30m)**
	Blood(CS)	5	5	5	4	5
	Brain(CS)[19]	8	8	8	7	8
	Liver(CS)	4	5	5	5	5
	Skin(CS)	5	5	5	5	5
	Ear (LL)	-	-	-	8	8
Homo sapiens *in vivo* n=118, i=62	**Total(CS)**	**28 (24-29 y)**	**-**	**32 (45-50 y)**	**28 (60-65 y)**	**30 (75-80 y)**
	Blood(CS)	15	-	17	15	15
	Skin(CS)	13	-	15	13	15
Homo sapiens *ex vivo* Fibroblasts n=47	**Total**	**15**	**6**	**6**	**6**	**14**
	BJ	3 (PD34)	-	-	-	3 (PD72)
	HFF[20]	3 (PD16)	3 (PD26)	3 (PD46)	3 (PD64)	3 (PD74)
	IMR-90	3 (PD31)	-	-	-	2 (PD57)
	MRC-5[20]	3 (PD32)	3 (PD42)	3 (PD52)	3 (PD62)	3 (PD72)
	Wi-38	3 (PD35)	-	-	-	3 (PD57)

Ageing-regulated gene sets (JenAge and external ageing data)

For our data, we determined differentially expressed genes between the young and old age groups directly from the gene expression counts using the R package DEseq2[21]. We determined differential expression between the young and one of the two old groups using standard parameters. We deemed a gene to be differentially expressed if DEseq2 yielded a FDR-corrected p value ≤ 0.05 between young and one of the two old age groups. Genes found to be differentially expressed in different directions between the young and the two old age groups were not considered further. For the five fibroblast cell lines, lists of genes differentially regulated in individual cell lines were merged to obtain a unified list since genes differentially expressed between different fibroblast cell lines have been reported to show a strong overlap[20]. Genes that showed discordant regulation between any two cell lines were discarded. For non-human data, genes were mapped to their human orthologues using BioMart[22].

For deriving ageing-regulated genes from the cross-species comparison, ageing-regulated processes were mapped to the genes constituting them by using the gene-to-process annotation used for deriving process activity from the gene expression data. Thus, we obtained a list of genes belonging to processes induced by ageing, yielding ageing-induced genes, and a list of genes belonging to processes repressed by ageing, yielding ageing-repressed genes. In the case where a gene was assigned to at least one process induced during ageing and at least one process repressed during ageing, the corresponding gene was discarded.

Moreover, we determined a list of consistently ageing-regulated genes across our ageing data sets based on the above lists by counting for each gene (or its human orthologue) the number of cases in which it was significantly down-regulated or up-regulated in the individual data sets (13 JenAge data sets). We ordered genes according to the absolute difference in number of cases of significant up- or down-regulation and considered those genes as consistently regulated in which this difference was at least five. Thus, we obtained a list of 896 genes with consistent ageing-associated regulation across species (Supplementary Data 1). For motif enrichment this gene list was tested against motif gene sets of transcription factors ("TFT – transcription factor targets") using mSigDB[23].

We complemented our data set with previously reported differentially expressed genes in various human tissues originating from blood (a cross-cohort study[24] and expression data from the San Antonio Family Heart Study[25]), muscle[26], brain[27] and breast[28]. Additionally, ageing-regulated gene sets for humans were obtained from the Digital Ageing Atlas[29] accessed on August 6, 2014.

Supplementary Table 3 Cross species ageing.

	Ageing data set	Genes repressed	Genes induced	Source
**CSA**	Gene Ontology	2131	4952	Our data
	KEGG Pathway	116	1449	Our data
	Metabolism	136	125	Our data
**Species ageing (JenAge data)**	*D. rerio* liver	461	412	Our data
	D. rerio brain	2899	2912	Our data
	D. rerio skin	1570	1829	Our data
	N. furzeri liver	1404	1227	Our data
	N. furzeri brain	3249	2952	Our data
	N. furzeri skin	3432	3352	Our data
	M. musculus liver	1028	1670	Our data
	M. musculus brain	748	965	Our data
	M. musculus skin	3792	2994	Our data
	M. musculus blood	1550	2174	Our data
	H. sapiens skin	992	1509	Our data
	H. sapiens blood	293	102	Our data
	H. sapiens fibroblasts	6385	7053	Our data
**External data**	*H. sapiens* blood (cross-cohort study)	889	595	[24]
	H. sapiens blood (SAFHS)	1858	1453	[25]
	H. sapiens muscle	280	326	[26]
	H. sapiens brain	599	586	[27]
	H. sapiens breast	277	405	[28]
	H. sapiens Digital Ageing Atlas	417	434	[29]

Disease data sets

Disease data sets were mostly obtained from Gene Expression Omnibus[30] (GEO) and the International Cancer Genome Consortium (ICGC)[31] for cancer related data. For each disease, GEO was searched with the respective term and data sets considered in order of the number of available samples. Except for three cancer data sets, studies were excluded from the analysis if no information about the age of the donors was available or healthy and disease samples were not age-matched. In some studies, age information was available and participants were not age-matched. In these cases we iteratively simultaneously removed the youngest sample from the controls and the oldest samples from the cases until the difference in median age between cases and controls was smaller than two years. For several major disease phenotypes, two data sets originating from the same tissue were included (in those cases the two largest studies in GEO fulfilling the inclusion criteria) to ensure that ageing-mediated disease alignment was not dataset-specific. Data originating from GEO was processed using the GEO2R script. Gene identifiers were translated to Ensembl gene IDs using BioMart[22].

For cancer data, read counts per transcript were downloaded from the ICGC Data Portal[32] for the following tissues: bladder, breast, colon/rectum, head/neck, kidney, liver, lung, ovary and prostate. Exclusively data without any publication limitation were used. The p values for differentially expressed genes were calculated with DeSeq2 v1.4.5[33] using methods based on (i) the estimation of size factors, which controls for differences in the library size of the sequencing experiment, (ii) the estimation of dispersion for each gene and (iii) negative binomial generalized linear models ("nbinomWaldTest"). To improve stability, a shrinkage estimation for dispersions and fold changes was applied. Outliers were detected and removed per transcript using the function "replaceOutliersWithTrimmedMean". Differential expression was determined considering the paired design in the sequencing data (i.e. healthy and disease tissue from the same patient). For the analysis of the skin cancer samples, a quantile normalization was applied on the Series Matrix File of the dataset GSE3189[34] from Gene Expression Omnibus. P values were determined using a Wilcoxon rank-sum test (R function ‘wilcox.test’) and corrected for multiple testing by controlling the false-discovery rate (FDR, Benjamini and Hochberg[35]). Details on the individual data sets are provided in Supplementary Table 2.

The potential influence of tumor cellularity was tested based on the renal cancer expression data (ICGC project code RECA-EU) for which information on tumor composition was provided. Data were split into samples with tumor cell content 41-60%, 41-80%, and 81-100%. Each batch was analyzed like described for the other cancer data sets. In total, 17,721 genes were significant differentially expressed between tumor and control in at least one group. Out of these genes, 15,105 genes had a fold change pointing in the same direction in all batches. Results of a pairwise comparison are shown in Supplementary Table 4.

Supplementary Table 4 Pairwise comparison between sample batches with different tumor cellularity. Exclusively genes that were significantly differentially expressed between tumor and control in at least one of the three batches (n=17,721) were considered in the comparison of fold-changes between batches.

Cellularity batch 1	Cellularity batch 2	Number of genes significant in first batch/second batch/both batches (tumor vs control)	Number of genes having same fold change direction in both batches (tumor vs control)
41-60	61-80	5,259 / 16,137 / 4,885	15,841 (89%)
41-60	81-100	5,259 / 10,969 / 4,050	15,319 (86%)
61-80	81-100	16,137 / 10,969 / 9,684	16,765 (95%)

Supplementary Table 5 Disease data sets used for the comparison of ageing and disease-associated gene regulation.

	Tissue/Disease	cases	controls	Reference	Comment
**Cancer**	Bladder	17	17	ICGC BLCA-US	Paired data
	Blood	1484	72	GEO GSE13204[36]	Not age-matched
	Brain	454	28	GEO GSE68848[37]	Not age-matched
	Breast	110	110	ICGC BRCA-US	Paired data
	Cancer proliferation index	60 cancer cell lines (NCI-60)		Correlation between gene expression and cancer growth rate in the NCI-60 panel[38]	
	Colon and rectum	47	47	ICGC COAD-US, READ-US	Paired data
	Head and neck	99	99	ICGC HNSC-US	Paired data
	Kidney	102	102	ICGC KIRC-US, KIRP-US	Paired data
	Liver	48	48	ICGC LIHC-US	Paired data
	Lung	99	99	ICGC LUAD-US, LUSC-US	Paired data
	Pancreas	45	45	GSE28735[39]	Paired data
**Cardiovascular diseases**	Coronary artery disease, plaque	32	32	GSE43292[40]	Paired data
	Coronary artery disease, blood	99	99	GSE20681[41]	
	Heart failure, artery, left ventricle	177	136	GSE57345[42]	
	Hypertension, blood	7017 individuals		Association between gene expression and blood pressure phenotypes based on model fit in a meta-analysis[43]	
	Myocardial infarction, peripheral blood mononuclear cells	111	46	GSE59867[44]	First day of admission vs. CAD controls
	Stroke, blood (Stroke - blood 1)	29	14	GSE16561[45]	Age-matched through sample removal (see above)
	Stroke, blood mononuclear cells (Stroke – blood 2)	20	20	GSE22255[46]	
**Type 2 diabetes**	Type 2 diabetes, pancreatic islets (diabetes - islets 1)	54	9	GSE38642[47]	
	Type 2 diabetes, pancreatic islets (diabetes - islets 2)	20	57	GSE41762[48]	
	Type 2 diabetes, liver	7	8	GSE14901[49]	
	Insulin resistance, blood	19	20	GSE20950[50]	
**Neurodeg. diseases**	Alzheimer's disease, brain tissue	176	181	GSE15222[51]	
	Alzheimer's disease, blood	49	67	GSE63060[52]	AD vs. healthy controls (case-control population)
	Mild cognitive impairment, blood	49	67	GSE63060[52]	MCI vs. healthy controls (case-control population)
	Parkinson's disease, blood (Parkinson – blood 1)	93	49	GSE57475[53]	
	Parkinson's disease, blood (Parkinson – blood 2)	50	22	GSE6613[54]	

Supplementary Note 3: Conservation of ageing-associated transcriptional regulation and randomization tests

Conservation of ageing-associated regulation

Since we observed many significant ageing-associated transcriptional changes for individual species, we tested whether the functional signature of ageing that we identified could be explained by a random overlap in ageing-regulated processes across the individual species that we considered. To this end, we randomly reordered the direction of change as well as its significance between the processes for each species and tissue. Thus, the number of significant ageing-associated transcriptional changes for the individual species and tissues remained the same. We repeated this process 1000 times and determined the functional signature of ageing for each repetition using the same criteria as in the main manuscript (i.e. a process is part of the functional signature of ageing if it is significantly regulated across all species and shows the same significant direction of change in at least one of the fish and one of the mammalian species).

In the randomization experiments, we observed that the number of cases of conserved regulation was usually at the same level as the number of cases with opposing regulation (Supplementary Fig. 6). In contrast, the number of conserved ageing-associated changes in the functional signature of ageing in our data was much higher and the number of opposing changes was much smaller than in the randomization experiments. This confirms that on a functional level there is a strong conservation of ageing-associated changes across species.

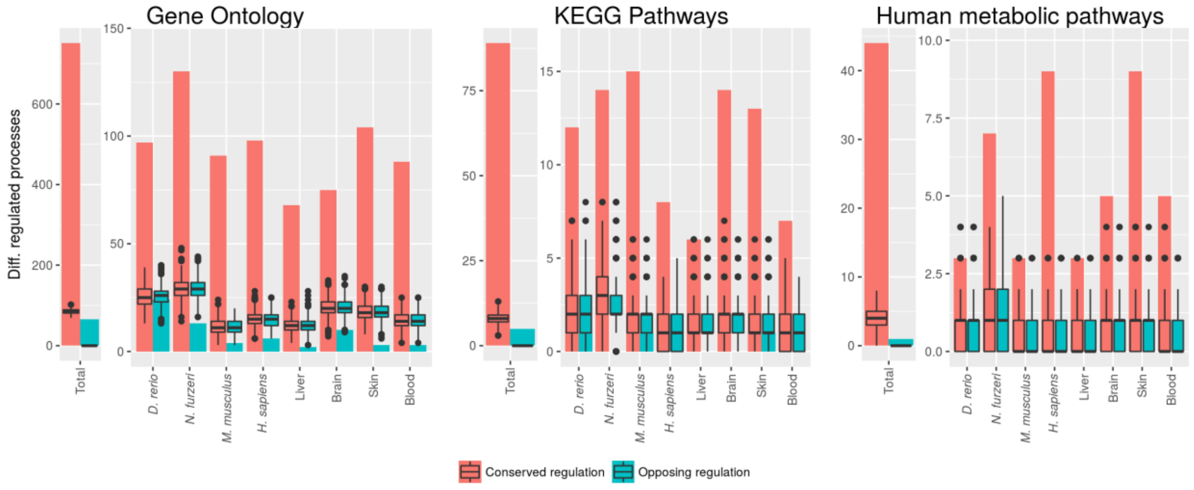

Supplementary Figure 6 Conserved and opposing regulation for randomly distributed significant ageing-associated changes. For each ontology the number of conserved ageing-associated changes and the number of opposing changes is displayed. For each subset of data (species or tissue), processes with conserved changes correspond to those processes with a significant ageing regulation in the same direction like the cross-species analysis. Likewise, processes with opposing regulation show a significant inverse regulation compared to the cross-species analysis. The left subplot for each ontology corresponds to the overall number of cases of conserved and opposing regulation across the individual species and tissues. The right subplot displays the number of conserved and opposing ageing-associated changes for individual species and tissues (as barplots). Boxplots denote the number of conserved and opposing changes for 1000 repetitions in which the association between processes and significant ageing-associated changes was randomly reordered.

Moreover, since previous works have only reported little overlap in the differential ageing-associated regulation of genes across species, we performed pairwise comparisons between ageing-regulated gene sets between all ageing data sets in our study. Thus, for each pair of aging data sets we determined the overlap in significantly ageing-regulated genes and the concordance of ageing

regulation (i.e. which fraction of common ageing-regulated genes shows the same direction of regulation). In general we found a very strong concordance in ageing-regulated gene sets also on the gene level (Supplementary Fig. **7**).

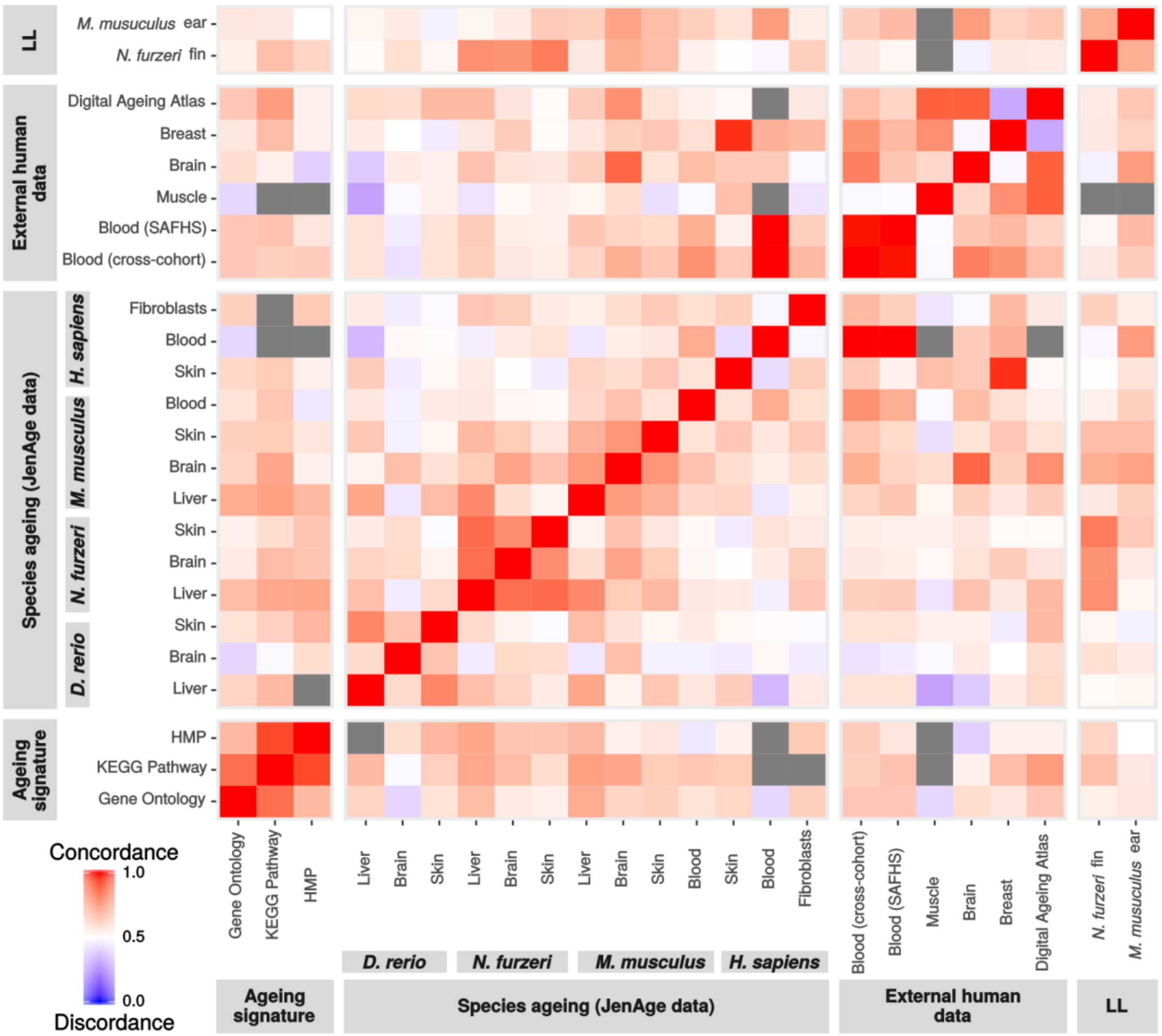

Supplementary Figure 7 Pairwise comparison of ageing-regulated gene sets. All ageing-regulated gene sets were compared between each other and the concordance of the direction of regulation was determined. Concordance refers to the fraction of genes that show the same direction of regulation during ageing in the two gene sets. A value of 1 indicates that all genes show the same direction of regulation while a value of 0 indicates a complete opposition. Grey areas indicate cases without significant overlap in ageing-regulated gene sets in the pairwise comparison based on a one-sided Fisher's exact test followed by correction for multiple testing across all comparisons.

Randomization of age assignments

We repeated the procedure for deriving ageing-regulated processes after randomly swapping the age labels of our samples. Swapping of age labels was performed within each tissue in each species separately, thus avoiding swapping age-labels between e.g. samples with different numbers of replicates. Subsequently, we determined ageing-regulated processes and performed the same filtering steps to obtain processes with significant ageing regulation across species (i.e. a process has to show a significant ageing regulation across species and in at least one of the mammalian as well as one of the fish species). We repeated the process 100 times and obtained no run with more than two significant ageing-regulated processes for gene ontology (various processes in each run). For KEGG, alpha linolenic acid metabolism was detected as significantly ageing regulated five times and for

human metabolic pathways fatty acid oxidation seven times as well as keratan sulfate synthesis four times.

We moreover conducted a randomization study of ageing-mediated disease alignment. To this end, for each ageing-regulated gene set, we randomly exchanged genes between the ageing-repressed and ageing-induced sets. Afterward we tested whether the randomly reassigned ageing-repressed or –induced genes showed a significant disease alignment as described in the main manuscript. After correcting p values for multiple testing in each run and performing 100 runs of the random resampling procedure, we did not observe a single instance of significant ageing-mediated disease alignment in the randomized gene sets.

Supplementary Note 4: Ageing-associated regulation of oncogenes and tumor suppressors

Comparison with lists of known oncogenes and tumor suppressors

We tested whether the frequency of oncogenes or tumor suppressors found among ageing-induced or ageing-repressed genes was significantly higher than expected, given the total number of genes annotated as oncogenes among genes covered by Gene Ontology (n=10.865) and KEGG Pathway (n=5.000), respectively, using a one-sided Fisher exact test. Depletion tests were performed similarly by determining whether the number of age-regulated oncogenes or tumor suppressors was significantly lower than expected. Differentially regulated processes were mapped to genes as described in Supplementary Note 2.

Supplementary Table 6 P values of enrichment and depletion analyses of oncogenes and tumor suppressors in ageing-induced and ageing-repressed genes. The first value in each cell denotes the p value of the test for enrichment and the second the test for depletion of oncogenes as well as tumor suppressors in process-derived ageing-regulated genes. Enrichment and depletion was tested using a one-sided Fisher exact test (see Supplementary Note 8 for details).

	**Gene Ontology**		**KEGG Pathway**	
	Ageing Repressed	Ageing Induced	Ageing Repressed	Ageing Induced
Oncogenes	$3.3*10^{-26}/1.0$	$1.0/2.0*10^{-13}$	0.75/0.50	$1.0/4.3*10^{-6}$
Tumor suppressors	$1.0/1.5*10^{-3}$	$1.0/4.2*10^{-3}$	0.75/0.5	$8.3*10^{-4}/1.0$

Analysis of somatic copy number variations in tumors

Genes with copy number variation (CNV) in human tumors (n=23 tumors, BLCA, BRCA, CESC, COAD, COADREAD, GBM, GBMLGG, HNSC, KIRC, KIRP, LAML, LGG, LIHC, LUAD, LUSC, OV, PRAD, SARC, SKCM, STAD, STES, THCA, UCEC) of 17 different tissues and a minimum number of 200 samples from The Cancer Genome Atlas (downloaded from http://gdac.broadinstitute.org/, Gistic2, Level 4, run 2015_08_21) were compared to ageing-induced and ageing-repressed genes for all three ontologies. We selected CNV genes which had a high level amplification (+2) or high level of deletion (-2) in at least five percent of all samples of a tumor entity. To select genes with CNVs that are of importance across different cancer types, we considered only those that showed CNVs in at least two different cancer types originating from different tissues or organs. Thus, we obtained a list of 5,532 amplified genes and 473 deletion genes.

Enrichment analysis using a one-sided Fisher's exact test revealed an enrichment of deletion genes in the ageing-induced gene set based on Gene Ontology and in KEGG Pathway (Supplementary Table 7). Moreover, amplified genes were depleted among ageing-induced genes in KEGG Pathway.

Supplementary Table 7 P values of enrichment and depletion analyses of amplified genes and deletion genes in ageing-induced and ageing-repressed genes. The first value in each cell denotes the p value of the test for enrichment and the second the test for depletion of amplified genes as well as deleted genes in process-derived ageing-regulated genes. Enrichment and depletion was tested using a one-sided Fisher exact test (see Supplementary Note 8 for details).

	**Gene Ontology**		**KEGG Pathway**	
	Ageing Repressed	Ageing Induced	Ageing Repressed	Ageing Induced
Ampl. genes	0.90/0.11	0.34/0.67	0.62/0.48	0.99/0.01
Deletion genes	0.91/0.13	$1.4*10^{-2}/0.99$	0.82/0.50	$4.68*10^{-4}/1.0$

Supplementary Note 5: Ageing-mediated disease alignment at middle age

While malignant transformation rates show a considerable decline with old age, we also observed periods of ageing in which they show strong increases. In particular, we observe an increase in malignant transformation rates across all cancer sites that occurs roughly in the age group between 30 and 45 years in humans that results in a doubling of median rates (Supplementary Fig. 8A). This increase is observable also when considering female and male gender separately (Supplementary Fig. 8B,C). Moreover, in this period external causes are replaced by diseases as principal causes of death (cf. Fig. 1A of the main manuscript), which is not due to a decline in the frequency of external causes of death which remain relatively constant in terms of frequency within each ageing cohort during this time (data not shown). Interestingly, this part of human lifespan has previously been associated with a transient period of intensive transcriptional alterations in human skin[55] and is also associated with considerable alterations in the human reproductive system in both sexes[56,57].

We tested whether ageing-mediated disease alignment scores in the corresponding age groups reflect this pronounced increases in malignant transformation rates. To this end, we used transcriptomic data from monocytes and T-cells of humans aged between 15 years and 55 years [58]. Overall, the study included 461 healthy participants. We separated the microarray data into four age groups: 15 – 19, 20 – 29, 30 – 39, 40 – 49 and 50 – 55 years of age. We compared gene expression between the individual age groups using GEO2R with the same approach described in Supplementary Note 2 (section “Disease data sets”) and used an unadjusted p-value of 0.01 as cut-off for determining ageing-regulated genes. Determining AMDA scores based on the ageing-regulated genes, we observed a strong ageing-associated alignment with cancer gene expression signatures between 20 to 29 and 30 to 39 years of age in monocytes as well as T-cells (Supplementary Fig. 8D). This pattern is also observable when considering male and female data separately (Supplementary Fig. 8D). For monocytes, we observe also an alignment with degenerative diseases between these age groups and a reversion in T-cells, which is particularly pronounced in the female data. Interestingly, risk factors for cardiovascular disease such as hypertension and atherosclerosis occur with lower frequency in females compared to males in these age groups while they are similar at older ages[59,60]. Please note that we obtain similar results when using an FDR-corrected p-value cut-off of 0.05, but at a lower temporal resolution for determining ageing-regulated genes during middle age (age groups 15 to 29 vs. 30 to 45 in monocytes and T-cells).

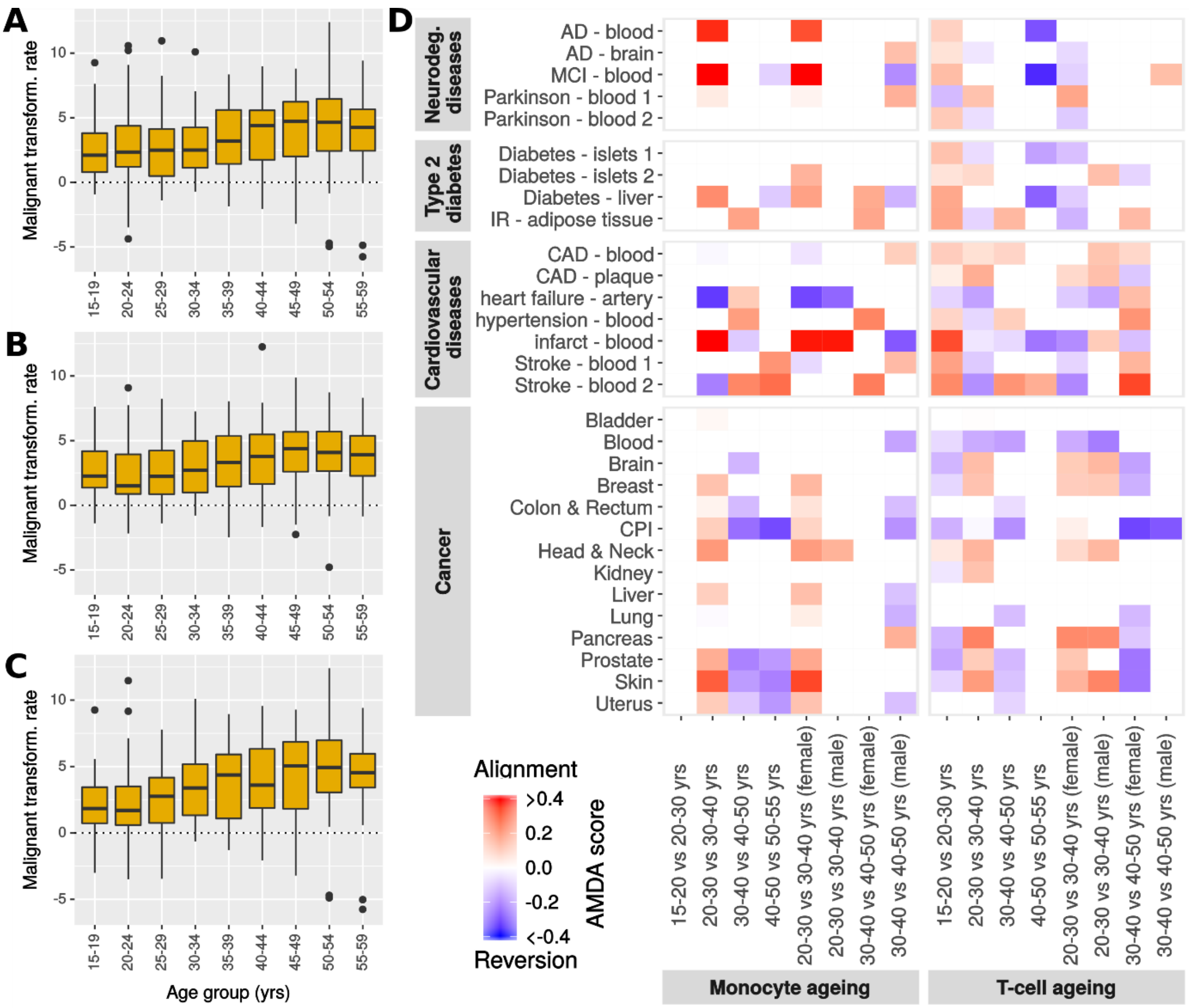

Supplementary Figure 8 Ageing-associated cancer epidemiology and disease alignment during middle age. **a-c** Malignant transformation rates across (a) both sexes as well as (b) females and (c) males separately from 15 to 59 years of age. **d** Ageing-mediated disease alignment for different age groups as well as gender (indicated in the labels) in gene expression data from monocytes and T-cells. Abbreviations: AD, Alzheimer's disease; CAD, coronary artery disease; CPI, cancer proliferation index; IR, insulin resistance; MCI, mild cognitive impairment; yrs, years.

Supplementary Note 6: Analysis of longitudinal data

To ascertain that the changes we observed also arise in individual organisms rather than being merely due to differences in the cohorts of individuals that survived to a specific age, we investigated whether we can observe the functional signature of ageing also during ageing of individuals. To this end, we determined longitudinal changes in processes belonging to the functional signature of ageing in fin clips of *N. furzeri* obtained from the same individuals at 10 and 20 weeks of age (n=45)[18] as well as in ear clips of the same mice obtained at 24 and 30 months of age (n=8). For each of these animals, the age of death was recorded. Thus, our analysis covered two age groups between which most individuals survived in *N. furzeri* (two mature age groups, Supplementary Figure 1 and Supplementary Note 2) and two age groups between which a considerable fraction of the population died (two old age groups in mice, Supplementary Figure 1 and Supplementary Note 2).

For longitudinal data, process activity was derived as described in the main manuscript without the rank normalization step since only data for a single species in a single tissue were compared. Only Gene Ontology processes were considered due to the larger number of significantly regulated processes compared to the other ontologies. To determine ageing-associated changes for each process, the average log fold-change across all individuals between the two ages of sampling was determined in the longitudinal data. For both species (*N. furzeri* and mice) only processes with significant ageing-associated regulation in the cross-sectional design between the youngest and the two oldest age groups were considered. For the comparison between longitudinal and cross-sectional ageing data, average fold-changes in process activity from longitudinal data were correlated with the difference in normalized process activity between young and the two old age groups in the cross-sectional data using Spearman correlation (Supplementary Fig. 9A). The lifespan-association of each process was determined as the Spearman correlation of log fold-changes in process activity between the two time points with the final lifespan of the corresponding individual. We found that changes of cross-sectional and longitudinal activity of processes belonging to the functional signature of ageing are significantly correlated in both species (Supplementary Fig. 9, Spearman rho=0.61, p value = 6.1×10^{-16} and Spearman rho=0.46, p value = 3.2×10^{-6} for *N. furzeri* and mice, respectively). Thus, the functional signature of ageing was also visible in the ageing of individual organisms, both in consecutive age groups with little age-related mortality and consecutive age groups with high mortality.

For the analysis of ageing mediated disease alignment in the longitudinal data, we used the 1000 genes with highest and 1000 genes with lowest log-fold change between the two age groups in *N. furzeri* and mice as ageing-induced and ageing-repressed genes, respectively. Results did not change qualitatively when considering a range of 500 to 2000 genes with highest or lowest fold-changes. Genes that had at least one sample without detectable expression were discarded. Subsequently, human genes were mapped to their closest orthologues in *N. furzeri* as well as mice and we repeated the tests of regulation of ageing diseases based on the up- and down-regulated genes from the longitudinal analysis.

The lifespan-association of each process was determined as the Spearman correlation of log fold-changes in process activity between the two time points of measurements with the time of death of the corresponding individual.

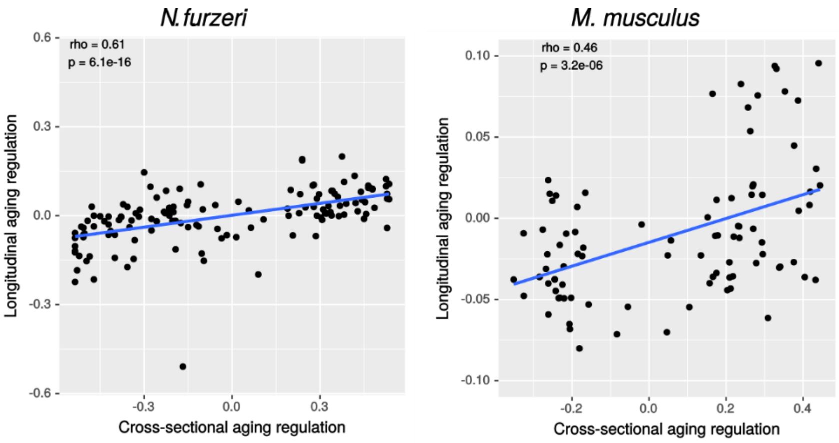

Supplementary Figure 9 Comparison of longitudinal and cross-sectional ageing. Ageing-associated regulation of processes in cross-sectional data is compared with longitudinal regulation of processes in *N. furzeri* and *M. musculus*. Cross-sectional regulation is based on the average difference in normalized process activity between the young and the two old age groups for all gene ontology processes that show a significant ageing-associated regulation in *N. furzer*i across all tissues. Longitudinal regulation is based on the average log fold-changes in process allocation between 10 and 20 weeks in fin clips (*N. furzeri*) and ear clips between 24 and 30 months of age (*M. musculus*). Dots correspond to individual processes and the blue line to the regression through all points. In the upper left corner of each plot, the Spearman correlation and its significance are provided.

Supplementary Note 7: Influence of cohort effects on ageing-mediated disease alignment in cross-sectional data

As an alternative approach to test whether cohort effects play a role in the observed relationship between ageing-associated regulation and the epidemiology of ageing-associated diseases, we repeated the analysis of the influence of ageing-associated changes on ageing diseases while excluding the second age-group of old organisms (“old$_2$”). Hence, we only considered the changes in process activity between the age groups young and old$_1$. For this old age group, most of the individuals were still alive and thus cohort-effects should be strongly reduced (cf. Supplementary Fig. 1). In high similarity of the analyses including the oldest age group, we found that ageing-associated gene expression changes opposed gene expression changes observed in cancer while gene expression changes of non-cancerous diseases were promoted.

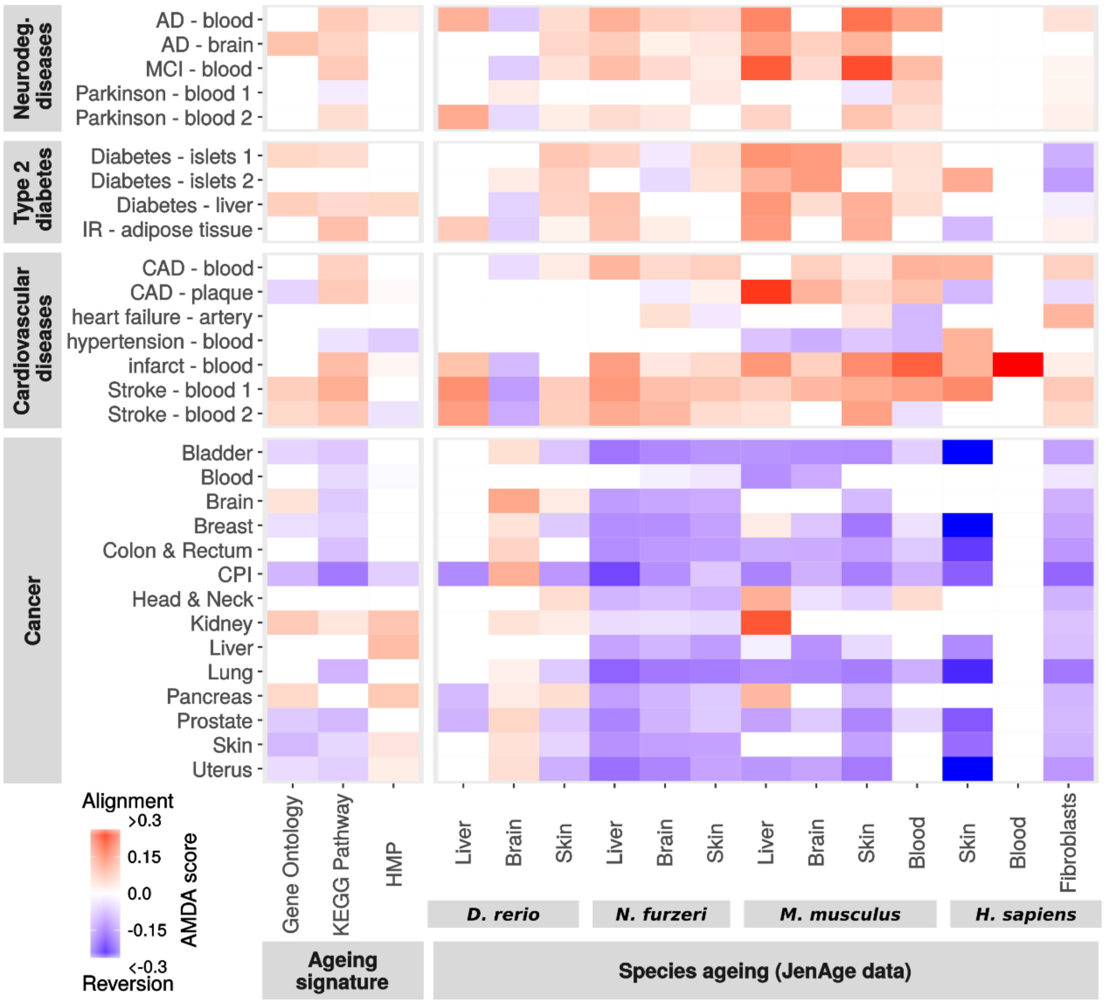

Supplementary Figure 10 Ageing-mediated disease alignment excluding old$_2$. Ageing-mediated disease alignment scores (AMDA scores) for different ageing data sets (x-axis) and diseases (y-axis). Diseases are grouped into the four main disease categories. Abbreviations: AD, Alzheimer’s disease; CAD, coronary artery disease; CPI, cancer proliferation index; HMP, human metabolic pathways; IR, insulin resistance; MCI, mild cognitive impairment.

Supplementary Note 8: Supplementary Methods

Influence of samples originating from the same animal

Since using RNA-seq data from different tissues of the same animal (see Supplementary Data 1, sheet 'Sample overview', for details) might affect our statistical analyses, tests for transcriptomic changes between the young and the two old age states were repeated 1000 times per ontology, each time sampling one measurement from each individual at random. Next, we tested each process using the ANOVA-based procedure described in the main manuscript. For Gene Ontology we found that out of 171 processes with significant ageing-associated transcriptional changes, 102 were significant in at least 500 random samples and 50 were significant in at least 900 of the 1000 random samples. For KEGG Pathway we found that out of 18 processes, 13 were significant in at least 500 random samples, 8 in at least 900 random samples. For Template Metabolism we found that out of 10 processes, 6 were significant in at least 500 random samples, 4 in at least 900 random samples. Only for Gene Ontology we found two processes ("response to estrogen", GO:0043627, p value of 0.073 in the cross-species analysis and "regulation of pathway-restricted SMAD protein phosphorylation",GO:0060393, p value of 0.065 in the cross-species analysis) that did not occur in any of the resamples. The number of times each process was found to be significantly associated with ageing is given in Supplementary Data 1. These results showed that the use of several tissues from the same animal only marginally influenced our results.

Sensitivity analysis of ageing regulated processes

We performed several sensitivity tests to assess the accuracy of ageing regulated processes that were identified.

We tested whether there was a considerable number of processes lost due to requiring that each process for each species and tissue contained at least five genes with detectable expression across all samples. For humans, we found 53 GO terms with at least 20 annotated genes that were not considered due to this requirement and only 12 with more than 50 genes which is rather small compared to the total number of 1563 considered processes (see Supplementary Data 1).

We repeated the analysis for a different approach of deriving process activity, whereby we first rank normalized all gene expression values for each tissue and each species to a range from 0 to 1 before determining process activity. Thus, each gene had the same weight when determining process activity. Determining significantly ageing-regulated processes, we found that 65 of the processes, which were significant in the summation-based derivation of process activity, were also significantly ageing-regulated based on normalized gene expression values. One process changed the direction of regulation. Overall, the functional categories of ageing-regulated processes were similar for both approaches (Supplementary Data 1).

Additionally, we investigated differential expression of genes within the processes, which were classified as ageing-regulated by determining for each process the frequency of pertaining genes with significant dysregulation across our ageing data sets. For Gene Ontology, 138 of 171 processes (81%) genes belonging to a process were also preferentially regulated in the direction of the regulation of the process (Supplementary Data 1).

Moreover, we determined ageing-regulated processes for the case in which all age groups were considered in the analysis for Gene Ontology. Out of the 523 processes, which passed the model assumptions of the ANOVA procedure, 333 showed an ageing-associated regulation with a FDR p-

value<0.1. In contrast, for the comparison between the young and the two old age groups we observed 448 processes with a FDR p-value<0.1 (out of 900 processes that passed the model assumptions). Among the top 50 age-regulated processes across all age groups, 49 were also age-regulated in the comparison between the young and the two old age groups (87 among the top 100). Thus, including all age groups yields a highly similar though smaller number of ageing-regulated processes. More information on this analysis is provided in Supplementary Data 2.

Ageing-mediated disease alignment (AMDA) scores

For each combination of disease *d* and ageing-regulated gene set set *a* consisting of sets of ageing-induced a_{UP} as well as ageing-repressed genes a_{DOWN}, we determined the AMDA score, $AMDA_{d,a}$, as follows. First, we determined for each disease data set fold-changes between case and control samples. Subsequently, fold-changes were quantile normalized across all diseases. Next, for each disease and each gene, we replaced fold-changes by their absolute ranks in the list of fold-changes multiplied with the corresponding direction of change (+1 if induced, -1 if repressed). Finally, fold-changes were divided by the number of genes covered by each disease data set such that fold-changes had a range from -1 to +1. $AMDA_{d,a}$ was then determined by subtracting the sum of normalized fold-changes of ageing-repressed genes from the sum of normalized fold-changes of ageing-induced genes and dividing by the total number of differentially regulated genes:

$$AMDA_{d,a} = \frac{\sum fc_d(a_{UP}) - \sum fc_d(a_{DOWN})}{|a_{UP}| + |a_{DOWN}|}$$

with $fc_d(\cdot)$ returning the normalized fold-changes of genes in the respective disease data set and $|\cdot|$ corresponding to the size of a gene set.

We assumed the AMDA score to be significant, if a Wilcoxon rank-sum test of normalized fold-changes of genes from a disease data sets between ageing-repressed and ageing-induced genes yielded a FDR-corrected p value < 0.05.

Disease alignment contribution (DAC) scores, process regulation and multi-dimensional scaling plots

We assessed the relevance of individual ageing-regulated processes in ageing-mediated disease alignment by the DAC score. The DAC score for a process *p* and a diseases category *C*, $DAC_{p,C}$ indicates how much the genes belonging to process *p* contribute to the ageing-mediated disease alignment for diseases belonging to the disease category *C*. Ageing-associated diseases were categorized into cancer, cardiovascular diseases, neurodegenerative diseases and type 2 diabetes as indicated in Fig. 3 of the main manuscript. We did not consider cancer proliferation indices as well as the hypertension data since they correspond to correlation-based analyses (while fold-changes of gene expression between cases and controls was available for the other disease data sets). For each disease category *d*, we normalized fold-changes in gene expression in the same way like for determining AMDA scores, but performed the rank normalization step just across genes measured across all data sets belonging to disease category *C*. For each disease $d \in C$ and each ageing-associated data set *a* consisting of sets of ageing-induced a_{UP} as well as ageing-repressed genes a_{DOWN}

(from the set of ageing-regulated gene sets *A*), we determined an additional AMDA score $AMDA_{d,a}^{\bar{p}}$ excluding fold-changes of genes belonging to process *p*, p_g

$$AMDA_{d,a}^{\bar{p}} = \frac{\sum fc_d(a_{UP} \setminus \mathrm{p_g}) - \sum fc_d(a_{DOWN} \setminus \mathrm{p_g})}{|a_{UP} \setminus \mathrm{p_g}| + |a_{DOWN} \setminus \mathrm{p_g}|}$$

with $fc_d(\cdot)$ returning the normalized fold-changes of genes in the respective disease data set and $|\cdot|$ corresponding to the size of a gene set. Additionally, we determined the normal AMDA score, $AMDA_{d,a}$ including genes belonging to *p* as described in the previous section. Please note, however, that $AMDA_{d,a}$ values determined for calculation of DAC scores might differ from those determined in the analysis of ageing-mediated disease alignment since we considered only genes for which fold-changes across all diseases in a disease category were available.

Moreover, we determined the significance in the shift of the p value after removing the genes belonging to a process $s_{p,d,a}$ by

$$s_{p,d,a} = -\log\left(wilcox\left(fc_d(a_{DOWN}), fc_d(a_{UP})\right)\right) + \log\left(wilcox\left(fc_d(a_{DOWN} \setminus \mathrm{p_g}), fc_d(a_{UP} \setminus \mathrm{p_g})\right)\right)$$

where "*wilcox(set₁,set₂)*" returns the p value of the corresponding test. In cases where $s_{p,d,a}$ was smaller than zero (i.e. AMDA scores were more significant after removing genes belonging to a process), it was set to zero.

A crude DAC score $cDAC_{p,C}$ for a process *p* and a diseases category *C* was then obtained by sum of shifts in disease alignment weighted by shifts in p values:

$$cDAC_{p,C} = \sum_{d \in C} \sum_{a \in A} \left(AMDA_{d,a} - AMDA_{d,a}^{\bar{p}}\right) \cdot s_{p,d,a}$$

Finally, the disease alignment score for each disease was obtained by dividing the crude DAC score with the number of comparisons considered (i.e. number of ageing data sets multiplied with number of disease data sets) and was normalized to a maximum absolute value of 1 across all disease categories.

Process fold-changes for ageing and disease categories were determined by combining raw fold-changes between cases and controls from the disease data sets with fold-changes from the ageing data sets. Fold-changes for genes in ageing data sets were determined for all genes that had orthologues across all four species considered in our analysis. Genes were mapped to their closest human orthologue using Ensembl's BioMart[22] and fold-changes determined between the youngest and the two oldest age groups. Subsequently, fold-changes were quantile normalized across all disease and ageing data sets and rank normalized using the same approach described for the DAC scores. Fold-changes of genes were averaged for the individual disease categories and ageing separately. Process fold-changes for ageing or a disease category were then obtained as the median of averaged normalized fold-changes of genes belonging to that process. Please note that ageing-associated process fold-changes are not necessarily the same compared to those obtained in the cross-species analysis since, for this analysis, a different approach for determining process activity

changes was used (based on the sum of gene expression values rather than the median of fold-changes). The approach applied in the cross-species comparison is only applicable to RNASeq-data and hence for comparison between ageing and diseases on the process level we used the same approach that was applicable to the mostly microarray-based disease data.

To obtain multi-dimensional scaling plots of disease/ageing-specific data sets, we either considered normalized fold-changes of genes (Fig. 4D of the main manuscript) or process fold-changes of the top 30 processes according to total DAC score across all disease categories (Fig. 4C of the main manuscript). Fold-changes were normalized as described above but only genes considered with fold-changes present in all ageing and disease data sets. To determine distances between all pairs of data sets (ageing and diseases), we correlated either gene or process fold-changes between all pairs of data sets using Spearman correlation. Correlation values (Spearman's rho) were transformed into distances by subtracting them from 1. Subsequently, multi-dimensional scaling plots were generated using the "cmdscale()" function of R on two dimensions.

Identification of shared risk loci

All risk alleles from published genome-wide association studies were obtained from GWAS catalog[62] release e87 dated January 4th, 2017. Among the reported risk variants, we filtered for those for which the identity of the risk allele as well as an odds ratio for the corresponding trait was provided. Thus, we did only consider binary traits (i.e. presence or absence of trait). We obtained a total of 8031 associations between SNPs and investigated traits. We identified risk variants for ageing-associated diseases by matching the reported trait for each genome-wide association study (GWAS) with a list of keywords/strings for each disease category as detailed in Supplementary Table 8.

Supplementary Table 8 Keywords for identification of disease categories.

**Cardiovascular diseases**	**Neurodegenerative diseases**	**Type 2 diabetes**	**Cancer**
Heart failure	Alzheimer	Type 2 diabet	Cancer
Stroke	Parkinson	Metabolic syndrome	Carcinoma
Atherosclerosis	Cognitive decline		Leukemia
Infarct	Dementia		Lymphoma
Hypertension	Amyloid deposition		Glioma
Aortic-valve calcification	Hyppocampal atrophy		Glioblastoma
Atrial fibrillation	Cerebral amyloid angiopathy		Melanoma
Coronary artery calcification	Cingulate cortical amyloid beta load		Myeloma
Cardio	Glaucoma		Meningioma
Coronary artery calcification	Macular degeneration		
Plaque			
Cardiac			

All reported disease terms for ageing diseases as well as other traits were manually checked for correct classification. Moreover, we removed associations with secondary traits such as e.g. mortality due to the individual diseases, associations with chemotherapy response or disease associations for patients carrying specific cancer-predisposing mutations (e.g. BRCA-mutation carriers). Moreover, we did not consider esophageal cancer since alcohol drinking is a strong risk factor for this cancer[63] and

heavy drinking is an established risk factor for cardiovascular diseases but often not controlled for[64]. Accounting for SNPs that were reported several times for a specific disease category, we obtained 729 associations for degenerative ageing diseases, 871 associations for cancer and 4112 associations for all other reported traits (excluding secondary traits for ageing-associated diseases or risk factors of ageing-associated diseases such as obesity). Please note that these other traits are mostly disease related (e.g. schizophrenia, inflammatory bowels disease, etc.).

In most cases, single nucleotide polymorphisms (SNPs) have a shared heritability with other polymorphisms in their genomic neighborhood. This shared heritability, referred to as linkage disequilibrium, leads to co-occurrence patterns of genetic polymorphisms in human populations. In genome-wide association studies, this mechanism leads to the problem that causative SNPs can often not be identified since they are in strong linkage disequilibrium with neighboring SNPs[65]. On the other hand, it is possible to impute the status of a SNP by just knowing the allele at another SNP that is in strong linkage disequilibrium with the query SNP. We used information on linkage disequilibrium to identify shared SNPs between cancer and degenerative diseases. To this end, we determined for all reported SNPs those with which they are in strong linkage disequilibrium ($r^2>0.7$) in at least two of the five major populations (African, American, European, East Asian, Southeast Asian) from the 1000 Genomes project (Phase 3, Oct. 2014, Genomes Project Consortium, et al. [66]) at a maximal distance of 1000 kbp using rAggr (http://raggr.usc.edu).

Given two sets of SNPs, e.g. from cancer and degenerative ageing diseases, we identified synergistic and antagonistic risk SNPs. Synergistic risk SNPs correspond to cases were the same SNP allele (i.e. one nucleotide of two existing variants) is either a risk allele for both disease types or there is a pair of risk alleles from the SNP sets of both diseases that is co-inherited through linkage disequilibrium according to the criteria stated above. Antagonistic risk SNPs correspond to cases where either alternative alleles at the same SNP position are contained as risk alleles in the two SNP sets or we identify pairs of SNPs from either sets that are in linkage disequilibrium but the co-inherited alleles between the two SNP sets correspond to alternative variants at the same genomic position. A full list of antagonistic and synergistic risk SNPs can be found in Supplementary Data 1.

We tested whether the number of synergistic and antagonistic risk SNPs between cancer and degenerative ageing diseases could also arise by chance or is typical for the overlap in genetic risk between cancer and other reported traits. First, we tested whether the number of antagonistic risk SNPs observed between cancer and degenerative ageing diseases could arise by chance based on shared risk SNPs between either disease type and other non-ageing associated traits reported in GWAS catalog. For cancer, we find 40 antagonistic and 50 synergistic risk SNPs with other traits (44.4% antagonistic risk SNPs). For degenerative ageing diseases, we find 23 antagonistic and 52 synergistic risk alleles with other traits (30.7% antagonistic risk SNPs). Based on these two frequencies we can test the likelihood of finding 36 antagonistic risk SNPs among 40 shared risk SNPs between cancer and degenerative ageing diseases using a binomial test (binomial test p-values of $1.99\cdot10^{-9}$ and $7.28\cdot10^{-15}$ tested against $p_0=0.444$ and $p_0=0.307$, respectively). Second, we performed randomization in which we compared the overlap between SNPs reported for both sets of diseases with sub-sampled SNPs reported for other traits contained in GWAS catalog. For the comparison involving cancer, we randomly selected the same number of 729 SNPs reported for degenerative ageing diseases from the set of 4112 SNPs reported for other traits. Then we determined synergistic and antagonistic risk SNPs between cancer SNPs and the randomly drawn SNP set. We repeated the procedure 10.000 times, similarly also for degenerative ageing diseases instead of cancer. We did not

encounter a single case with an equal or higher number of antagonistic risk alleles that we observed in the comparison between cancer and degenerative ageing diseases (36 antagonistic risk alleles). The maximal number of antagonistic risk SNPs observed in the randomization experiments was 19 for cancer and 17 for degenerative diseases. Considering the combined number of synergistic and antagonistic risk SNPs, there were no instances for cancer and five of 10.000 instances for degenerative diseases in which an equal or higher number of shared risk SNPs like in the comparison between cancer and degenerative diseases (40 shared risk SNPs) was detected. Thus, beyond the number of antagonistic risk SNPs between cancer and degenerative ageing diseases that is unlikely to have arisen by chance, the overlap in risk SNPs between cancer and degenerative diseases is also much larger than between either type of disease and other traits reported in GWAS catalog.

To test the robustness of the comparison of risk SNPs between cancer and degenerative ageing diseases, we repeated our analysis for different parameter choices including

1) requiring a stronger linkage disequilibrium (LD) between SNPs of $r^2 \geq 0.9$ for identifying SNPs with shared heritability
2) requiring linkage disequilibrium in at least three of the five populations of the 1000 genomes project
3) requiring a stronger reported p-value cut-off for reported SNPs of $p \leq 5*10^{-8}$
4) defining a minimal odds ratio cut-off of 1.1

For all analyses, we observed a much larger number of antagonistic risk SNPs compared to synergistic risk SNPs (Supplementary Table 9). Interestingly, more conservative parameters had stronger effects on synergistic risk SNPs with none remaining if requiring a more conservative p-value cut-off of less than $5*10^{-8}$ or a minimal odds ratio of 1.1. This suggests that the antagonism between cancer and degenerative ageing diseases is even more pronounced for more conservative cut-offs.

Moreover, we tested the effect sizes of shared risk alleles between cancer and degenerative diseases. We found that odds ratios between both disease categories were comparable and on average 1.24 (+/- 0.22) for degenerative diseases and 1.19 (+/- 0.09) for cancer (see Supplementary Data 1 for a detailed list of odds ratios).

Supplementary Table 9 Sensitivity analysis of number of synergistic and antagonistic risk alleles. The first column provides the parameter tested, the second column the number of remaining SNPs for degenerative ageing diseases (fulfilling the corresponding criteria), the third column the number of reported SNPs for cancer (fulfilling the corresponding criteria) and the last two columns the number of synergistic as well as antagonistic risk SNPs. Abbreviations: DAD, degenerative ageing diseases.

	**Total SNPs DAD**	**Total SNPs cancer**	**Synergistic risk SNPs**	**Antagonistic risk SNPs**
Full comparison	729	846	4	36
Strong LD ($r^2 \geq 0.9$)	729	846	1	17
LD in ≥3 populations	729	846	2	27
SNP p-value $\leq 5*10^{-8}$	417	557	0	26
Odds ratio ≥ 1.1	585	754	0	24

References

1. Rocca, W.A. *et al.* Trends in the incidence and prevalence of Alzheimer's disease, dementia, and cognitive impairment in the United States. *Alzheimers Dement* **7**, 80-93 (2011).
2. Driver, J.A., Djousse, L., Logroscino, G., Gaziano, J.M. & Kurth, T. Incidence of cardiovascular disease and cancer in advanced age: prospective cohort study. *BMJ* **337**, a2467 (2008).
3. Thunander, M. *et al.* Incidence of type 1 and type 2 diabetes in adults and children in Kronoberg, Sweden. *Diabetes Res Clin Pract* **82**, 247-55 (2008).
4. Field, A.E. *et al.* Impact of overweight on the risk of developing common chronic diseases during a 10-year period. *Arch Intern Med* **161**, 1581-6 (2001).
5. Cowie, C.C. *et al.* Full accounting of diabetes and pre-diabetes in the U.S. population in 1988-1994 and 2005-2006. *Diabetes Care* **32**, 287-94 (2009).
6. McAllister, E.J. *et al.* Ten putative contributors to the obesity epidemic. *Crit Rev Food Sci Nutr* **49**, 868-913 (2009).
7. Fishman, E.I., Stokes, A. & Preston, S.H. The dynamics of diabetes among birth cohorts in the US. *Diabetes care* **37**, 1052-1059 (2014).
8. Stovring, H. & Wang, M.C. A new approach of nonparametric estimation of incidence and lifetime risk based on birth rates and incident events. *BMC Med Res Methodol* **7**, 53 (2007).
9. Hanson, H.A., Smith, K.R., Stroup, A.M. & Harrell, C.J. An age–period–cohort analysis of cancer incidence among the oldest old, Utah 1973–2002. *Population studies* **69**, 7-22 (2015).
10. McEwen, L.N. *et al.* Diabetes reporting as a cause of death results from the Translating Research Into Action for Diabetes (TRIAD) study. *Diabetes care* **29**, 247-253 (2006).
11. Yashin, A.I. *et al.* Maintaining physiological state for exceptional survival: What is the normal level of blood glucose and does it change with age? *Mechanisms of ageing and development* **130**, 611-618 (2009).
12. Armitage, P. & Doll, R. The age distribution of cancer and a multi-stage theory of carcinogenesis. *Br J Cancer* **91**, 1983-9 (2004).
13. Frank, S.A. Age-specific acceleration of cancer. *Current Biology* **14**, 242-246 (2004).
14. Cairns, T. The ED01 study: introduction, objectives, and experimental design. *J Environ Pathol Toxicol* **3**, 1-7 (1980).
15. Pompei, F., Polkanov, M. & Wilson, R. Age distribution of cancer in mice: the incidence turnover at old age. *Toxicol Ind Health* **17**, 7-16 (2001).
16. Harding, C., Pompei, F. & Wilson, R. Corrections to: ''Age distribution of cancer in mice''. *Toxicol Ind Health* **27**, 265-70 (2011).
17. Reichwald, K. *et al.* Insights into Sex Chromosome Evolution and Aging from the Genome of a Short-Lived Fish. *Cell* **163**, 1527-38 (2015).
18. Baumgart, M. *et al.* Longitudinal RNA-seq analysis of vertebrate aging identifies mitochondrial complex I as a small-molecule-sensitive modifier of lifespan *Cell Systems* **2**, 122-132 (2016).
19. Frahm, C. *et al.* Transcriptional profiling reveals protective mechanisms in brains of long-lived mice. *Neurobiol Aging* **52**, 23-31 (2016).
20. Marthandan, S. *et al.* Similarities in gene expression profiles during in vitro aging of primary human embryonic lung and foreskin fibroblasts. *Biomed Res Int* **2015**, 731938 (2015).
21. Love, M.I., Huber, W. & Anders, S. Moderated estimation of fold change and dispersion for RNA-seq data with DESeq2. *Genome Biol* **15**, 550 (2014).
22. Smedley, D. *et al.* The BioMart community portal: an innovative alternative to large, centralized data repositories. *Nucleic Acids Res* (2015).
23. Subramanian, A. *et al.* Gene set enrichment analysis: a knowledge-based approach for interpreting genome-wide expression profiles. *Proc Natl Acad Sci U S A* **102**, 15545-50 (2005).
24. Peters, M.J. *et al.* The transcriptional landscape of age in human peripheral blood. *Nat Commun* **6**, 8570 (2015).
25. Kent, J.W., Jr. *et al.* Genotypexage interaction in human transcriptional ageing. *Mech Ageing Dev* **133**, 581-90 (2012).

26. Welle, S., Brooks, A.I., Delehanty, J.M., Needler, N. & Thornton, C.A. Gene expression profile of aging in human muscle. *Physiol Genomics* **14**, 149-59 (2003).
27. Berchtold, N.C. *et al.* Gene expression changes in the course of normal brain aging are sexually dimorphic. *Proc Natl Acad Sci U S A* **105**, 15605-10 (2008).
28. Pirone, J.R. *et al.* Age-associated gene expression in normal breast tissue mirrors qualitative age-at-incidence patterns for breast cancer. *Cancer Epidemiol Biomarkers Prev* **21**, 1735-44 (2012).
29. Craig, T. *et al.* The Digital Ageing Atlas: integrating the diversity of age-related changes into a unified resource. *Nucleic acids research*, gku843 (2014).
30. Barrett, T. *et al.* NCBI GEO: mining tens of millions of expression profiles--database and tools update. *Nucleic Acids Res* **35**, D760-5 (2007).
31. International Cancer Genome Consortium *et al.* International network of cancer genome projects. *Nature* **464**, 993-8 (2010).
32. Zhang, J. *et al.* International Cancer Genome Consortium Data Portal--a one-stop shop for cancer genomics data. *Database (Oxford)* **2011**, bar026 (2011).
33. Anders, S. & Huber, W. Differential expression analysis for sequence count data. *Genome Biol* **11**, R106 (2010).
34. Talantov, D. *et al.* Novel genes associated with malignant melanoma but not benign melanocytic lesions. *Clin Cancer Res* **11**, 7234-42 (2005).
35. Benjamini, Y. & Hochberg, Y. Controlling the false discovery rate: a practical and powerful approach to multiple testing. *Journal of the Royal Statistical Society. Series B (Methodological)*, 289-300 (1995).
36. Haferlach, T. *et al.* Clinical utility of microarray-based gene expression profiling in the diagnosis and subclassification of leukemia: report from the International Microarray Innovations in Leukemia Study Group. *J Clin Oncol* **28**, 2529-37 (2010).
37. Madhavan, S. *et al.* Rembrandt: helping personalized medicine become a reality through integrative translational research. *Mol Cancer Res* **7**, 157-67 (2009).
38. Waldman, Y.Y., Geiger, T. & Ruppin, E. A genome-wide systematic analysis reveals different and predictive proliferation expression signatures of cancerous vs. non-cancerous cells. *PLoS Genet* **9**, e1003806 (2013).
39. Zhang, G. *et al.* Integration of metabolomics and transcriptomics revealed a fatty acid network exerting growth inhibitory effects in human pancreatic cancer. *Clin Cancer Res* **19**, 4983-93 (2013).
40. Ayari, H. & Bricca, G. Identification of two genes potentially associated in iron-heme homeostasis in human carotid plaque using microarray analysis. *J Biosci* **38**, 311-5 (2013).
41. Elashoff, M.R. *et al.* Development of a blood-based gene expression algorithm for assessment of obstructive coronary artery disease in non-diabetic patients. *BMC Med Genomics* **4**, 26 (2011).
42. Liu, Y. *et al.* RNA-Seq identifies novel myocardial gene expression signatures of heart failure. *Genomics* **105**, 83-9 (2015).
43. Huan, T. *et al.* A meta-analysis of gene expression signatures of blood pressure and hypertension. *PLoS Genet* **11**, e1005035 (2015).
44. Maciejak, A. *et al.* Gene expression profiling reveals potential prognostic biomarkers associated with the progression of heart failure. *Genome Med* **7**, 26 (2015).
45. Barr, T.L. *et al.* Genomic biomarkers and cellular pathways of ischemic stroke by RNA gene expression profiling. *Neurology* **75**, 1009-14 (2010).
46. Krug, T. *et al.* TTC7B emerges as a novel risk factor for ischemic stroke through the convergence of several genome-wide approaches. *J Cereb Blood Flow Metab* **32**, 1061-72 (2012).
47. Taneera, J. *et al.* A systems genetics approach identifies genes and pathways for type 2 diabetes in human islets. *Cell Metab* **16**, 122-34 (2012).
48. Mahdi, T. *et al.* Secreted frizzled-related protein 4 reduces insulin secretion and is overexpressed in type 2 diabetes. *Cell Metab* **16**, 625-33 (2012).

49. Kirchner, H. *et al.* Altered DNA methylation of glycolytic and lipogenic genes in liver from obese and type 2 diabetic patients. *Mol Metab* **5**, 171-83 (2016).
50. Hardy, O.T. *et al.* Body mass index-independent inflammation in omental adipose tissue associated with insulin resistance in morbid obesity. *Surg Obes Relat Dis* **7**, 60-7 (2011).
51. Webster, J.A. *et al.* Genetic control of human brain transcript expression in Alzheimer disease. *Am J Hum Genet* **84**, 445-58 (2009).
52. Sood, S. *et al.* A novel multi-tissue RNA diagnostic of healthy ageing relates to cognitive health status. *Genome Biol* **16**, 185 (2015).
53. Locascio, J.J. *et al.* Association between alpha-synuclein blood transcripts and early, neuroimaging-supported Parkinson's disease. *Brain* **138**, 2659-71 (2015).
54. Scherzer, C.R. *et al.* Molecular markers of early Parkinson's disease based on gene expression in blood. *Proc Natl Acad Sci U S A* **104**, 955-60 (2007).
55. Haustead, D.J. *et al.* Transcriptome analysis of human ageing in male skin shows mid-life period of variability and central role of NF-kappaB. *Sci Rep* **6**, 26846 (2016).
56. Navot, D. *et al.* Poor oocyte quality rather than implantation failure as a cause of age-related decline in female fertility. *Lancet* **337**, 1375-7 (1991).
57. Hassan, M.A. & Killick, S.R. Effect of male age on fertility: evidence for the decline in male fertility with increasing age. *Fertil Steril* **79 Suppl 3**, 1520-7 (2003).
58. Raj, T. *et al.* Polarization of the effects of autoimmune and neurodegenerative risk alleles in leukocytes. *Science* **344**, 519-23 (2014).
59. Daugherty, S.L. *et al.* Age-dependent gender differences in hypertension management. *J Hypertens* **29**, 1005-11 (2011).
60. Joakimsen, O., Bonaa, K.H., Stensland-Bugge, E. & Jacobsen, B.K. Age and sex differences in the distribution and ultrasound morphology of carotid atherosclerosis: the Tromso Study. *Arterioscler Thromb Vasc Biol* **19**, 3007-13 (1999).
61. Tacutu, R. *et al.* Human Ageing Genomic Resources: integrated databases and tools for the biology and genetics of ageing. *Nucleic Acids Res* **41**, D1027-33 (2013).
62. Welter, D. *et al.* The NHGRI GWAS Catalog, a curated resource of SNP-trait associations. *Nucleic Acids Res* **42**, D1001-6 (2014).
63. Castellsague, X. *et al.* Independent and joint effects of tobacco smoking and alcohol drinking on the risk of esophageal cancer in men and women. *Int J Cancer* **82**, 657-64 (1999).
64. Rehm, J., Sempos, C.T. & Trevisan, M. Alcohol and cardiovascular disease--more than one paradox to consider. Average volume of alcohol consumption, patterns of drinking and risk of coronary heart disease--a review. *J Cardiovasc Risk* **10**, 15-20 (2003).
65. Zuk, O., Hechter, E., Sunyaev, S.R. & Lander, E.S. The mystery of missing heritability: Genetic interactions create phantom heritability. *Proc Natl Acad Sci U S A* **109**, 1193-8 (2012).
66. Genomes Project Consortium *et al.* A global reference for human genetic variation. *Nature* **526**, 68-74 (2015).
67. Kunstyr, I. & Leuenberger, H.G. Gerontological data of C57BL/6J mice. I. Sex differences in survival curves. *J Gerontol* **30**, 157-62 (1975).
68. Gerhard, G.S. *et al.* Life spans and senescent phenotypes in two strains of Zebrafish (Danio rerio). *Exp Gerontol* **37**, 1055-68 (2002).
69. Tozzini, E.T. *et al.* Parallel evolution of senescence in annual fishes in response to extrinsic mortality. *BMC Evol Biol* **13**, 77 (2013).
70. Campisi, J. Aging, cellular senescence, and cancer. *Annu Rev Physiol* **75**, 685-705 (2013).
