# Supplementary material for: Transcriptomic alterations during ageing reflect the shift from cancer to degenerative diseases in the elderly
Source: Nat Commun. 2018 Jan 30;9:327. doi: 10.1038/s41467-017-02395-2 (PMC5790807; doi:10.1038/s41467-017-02395-2)
Supplement: Supplementary file 3 — Description of Additional Supplementary Files [file 41467_2017_2395_MOESM3_ESM.pdf]

Description of Supplementary Files

File Name: Supplementary Data 1
Description: Information about samples, data on the multi-species comparison for processes that were identified as being consistently changed across species, AMDA-scores as well as DAC scores, data of the longitudinal analysis and shared risk SNPs between ageing diseases.

File Name: Supplementary Data 2
Description: Information about ageing-associated transcriptional changes for all considered processes, differentially regulated genes for ageing as well as disease analyses, disease alignment analyses.
